# Social Transmission of Experience of Agency: An Experimental Study

**DOI:** 10.3389/fpsyg.2016.01315

**Published:** 2016-08-30

**Authors:** Nima Khalighinejad, Bahador Bahrami, Emilie A. Caspar, Patrick Haggard

**Affiliations:** ^1^Institute of Cognitive Neuroscience, University College LondonLondon, UK; ^2^Consciousness, Cognition and Computation Group (CO3), Center for Research in Cognition and Neurosciences, ULB Neuroscience Institute, Université libre de BruxellesBruxelles, Belgium

**Keywords:** sense of agency, intentional binding, action observation, robotic hand, social context

## Abstract

The sense of controlling one’s own actions is fundamental to normal human mental function, and also underlies concepts of social responsibility for action. However, it remains unclear how the wider social context of human action influences sense of agency. Using a simple experimental design, we investigated, for the first time, how observing the action of another person or a robot could potentially influence one’s own sense of agency. We assessed how observing another’s action might change the perceived temporal relationship between one’s own voluntary actions and their outcomes, which has been proposed as an implicit measure of sense of agency. Working in pairs, participants chose between two action alternatives, one rewarded more frequently than the other, while watching a rotating clock hand. They judged, in separate blocks, either the time of their own action, or the time of a tone that followed the action. These were compared to baseline judgements of actions alone, or tones alone, to calculate the perceptual shift of action toward outcome and vice versa. Our design focused on how these two dependent variables, which jointly provide an implicit measure of sense of agency, might be influenced by observing another’s action. In the observational group, each participant could see the other’s actions. Multivariate analysis showed that the perceived time of action and tone shifted progressively toward the actual time of outcome with repeated experience of this social situation. No such progressive change occurred in other groups for whom a barrier hid participants’ actions from each other. However, a similar effect was observed in the group that viewed movements of a human-like robotic hand, rather than actions of another person. This finding suggests that observing the actions of others increases the salience of the external outcomes of action and this effect is not unique to observing human agents. Social contexts in which we see others controlling external events may play an important role in mentally representing the impact of our own actions on the external world.

## Introduction

The feeling of control over one’s own actions is referred to as ‘sense of agency.’ Sense of agency is not only fundamental to instrumental, goal-directed actions at the individual level, but also forms a cornerstone of everyday social life. In social animals, an instrumental action of one agent will have outcomes that affect other agents, so some social management of individual action is required. In particular, the sociolegal concept that each individual is responsible for their actions, and must answer for them to others, requires that individuals are first *aware* of their actions, and of the outcomes of those actions (Moretto et al., 2011; Frith, 2014). The relation between instrumental agency at the individual level and the social aspects of agency has rarely been explored experimentally: the present paper represents a first step in this direction.

At heart, sense of agency depends on a mental association between an intentional action and its sensory outcome (Spengler et al., 2009; Moore et al., 2011). Indeed, human sense of agency has several parallels with instrumental learning in animals (Rescorla and Wagner, 1972). In particular, both sense of agency and instrumental learning depend on a cognitive mechanism that represents contingent relation between action and outcome, based on previous experience (Cravo et al., 2009; Moore et al., 2009), and then computes predictions of an outcome, given an action (Frith et al., 2000; Wolpert and Ghahramani, 2000). The human experimental literature has focussed largely on the signals and conditions that produce a sense of agency. Relatively few human studies have focussed on how observation of others may influence the development of a sense of agency (Engbert et al., 2007).

In classical instrumental learning, animals are thought to learn instrumental relations by exploring the environment, and by trial and error (Thorndike, 1905). However, action-outcome association is acquired not only through individual learning, but also by observing others (Brass and Heyes, 2005; Burke et al., 2010). Imitation (Topál et al., 2008), and participation in joint action (van der Wel et al., 2012) appear to facilitate emergence of a sense of agency in social contexts, although neither is essential. Interestingly, the idea of learning by observation is central to most systems of education. On the other hand, we frequently interact with machines. Such artificial agents also perform functional actions which produce environmental outcomes. However, several studies on joint actions highlighted the role of human partnership (Sebanz et al., 2006; Obhi and Sebanz, 2011; Stenzel et al., 2012), which depends on creating a mental representation of the partner’s intentions (Sebanz et al., 2006). One of the established methods to study joint action is the joint Simon paradigm (Simon, 1969). In the standard Simon task, spatial match between stimulus and response facilitates task performance. Interestingly, the same effect is observed across two participants in a joint Simon task when the assigned stimulus corresponds to the response key that the individual is responsible for operating it (Sebanz et al., 2003; Dolk et al., 2014). This suggests that human agents share a mental representation with other co-actors. However, this shared representation diminishes when interacting with a non-human agent (Müller et al., 2011a) or dissimilar human co-actor (Müller et al., 2011b). A partner who is not recognized as sharing the same mental representations as ours, for instance a robot, would presumably not benefit from the “intentional stance” (Dennett, 1989). The effects of intentional vs non-intentional partners on action representation and sense of agency have not been systematically compared.

Several studies have reported effects of social context on measures of sense of agency. Indeed, the dominant experimental paradigms for explicit judgment of one’s own agency generally involve attributing an action (Sirigu et al., 1999) or an outcome to oneself, or to another agent (Wegner and Wheatley, 1999; Strother et al., 2010; Obhi and Hall, 2011a,b). However, explicit judgments of agency are rare in real life, and could vary because of interpretational factors such as social acceptability, or other socially mediated effects. Therefore, implicit measures may be more appropriate for elucidating socially mediated changes in the low-level experience of one’s own agency.

One suitable measure of the sense of agency is the perceived temporal relationship between a voluntary action and its sensory outcome (Haggard et al., 2002b). The perceived time of voluntary actions and their sensory consequences are attracted toward each other, relative to a baseline condition in which either action or outcome occurs alone. This ‘intentional binding’ does not occur for involuntary movements, or for associations between external events that do not involve voluntary actions (Cravo et al., 2009). Thus, intentional binding may tap into important low-level mechanisms of voluntary control over instrumental actions. At the same time, intentional binding was also reported to be sensitive to high-level factors such as social context. For example, perceived social power relations and social hierarchies modulate this effect (Obhi and Hall, 2011a,b; Poonian and Cunnington, 2013). In one recent study (Pfister et al., 2014), social interaction between two people was specifically constructed as part of the experimental design. One participant (the follower) made actions in response to the actions of another (the leader). Interestingly, the leader showed intentional binding both for her own actions and for the follower’s.

In the present experiment, we used intentional binding to investigate whether observing the actions of a human or a non-human agent could potentially influence one’s own sense of agency. Our design goes beyond previous studies in three important respects. First, we used implicit rather than explicit measures of sense of agency. Second, we organized a situation where the other’s action was clearly comparable to one’s own performance. Third, our hypothesis directly targeted the social modulation of action representation. We focused on the progressive emergence of sense of agency provided by ongoing social information, rather than on simply comparing pre-test and post-test phases. In order to test this hypothesis, we focused on changes across time of intentional binding between action and outcome. We hypothesized that sense of agency, as measured by intentional binding, would be affected by observing the actions of others. Specifically, the social context would potentiate the development of sense of agency, compared to a non-social task involving individual performance. To investigate whether these processes are unique to observing human agents, we additionally included a condition in which participants watched a non-human, robotic agent, performing similar actions. We further hypothesized that acquisition of sense of agency would be accordingly reduced in these conditions.

## Materials and Methods

### Participants

Seventy two healthy volunteers (21 males), aged 18–35 (mean 23 years) were recruited form UCL Institute of Cognitive Neuroscience data base. The study was approved by the UCL Research Ethics Committee, and conformed to the Declaration of Helsinki. All participants were right handed and had normal or corrected to normal vision and hearing. Those participants who were invited in pairs did not know each other before the experimental session.

### Behavioral Task

The intentional binding paradigm was used as a proxy measure of sense of agency, broadly following methods described elsewhere (Haggard et al., 2002b). Briefly, in each session, participants were seated in front of a computer screen (viewing distance: 60 cm) and fixated on a clock with single rotating hand. Each full rotation lasted 2560 ms. During the rotation, participants pressed the left or right arrow on a keyboard, at a time of their own free choice. After the key press, the clock hand stopped at a random location, participants made a time judgment according to condition (see later). Each experimental session consisted of six types of trials, presented in separate blocks, performed in a different randomized order by each participant. At the beginning of each block, brief instructions for the relevant condition were displayed on the screen. In the *baseline action left* and *baseline action right* conditions, participants had to press the left or right arrow, respectively, at a time of their own free choice. The clock hand stopped after 1500–2500 ms (at random), and participants then judged the clock hand position at the time of their key press, entering their response on the keyboard. In this condition, the participant’s actions produced no sensory outcome. In the *baseline beep* and *baseline buzz* conditions, participants were instructed to look at the clock but not to press any key. While the clock was rotating, a beep (2000 Hz, 100 ms duration), or a buzz (500 Hz, 100 ms duration), in separate blocks, was played over a loudspeaker, 1750–4000 ms (at random) after the onset of the trial. Participants were then asked to judge the clock hand position at the time of the tone. The timings were chosen to approximate the times of voluntary actions in other conditions.

In the *operant action* condition, participants pressed the left or right arrow key at a time of their own choosing. One key caused a beep with probability of 0.7 and the other key with probability of 0.3 after 250 ms. On the remaining trials, the buzz was played instead of the beep. The mapping of keys to sounds changed after every 9–11 trials, with the run length being random within these limits. Participants were instructed to choose the key that most often produced a beep. They were reminded that the mappings were never 100% for both key, and that from time to time, the outcomes associated with each key would switch, so it was important that they pay attention to the tones, and always choose the key that was most likely to give a beep. The theoretical motivation for using a probabilistic action-outcome relationship was to keep participants alert, interested and ready to learn throughout the experiment. Variable reward schedules require continuous attention, and make the occasional reward worth pursuing (Zeiler, 1968; Mazur, 1986; Bateson and Kacelnik, 1995).

**FIGURE 1:**
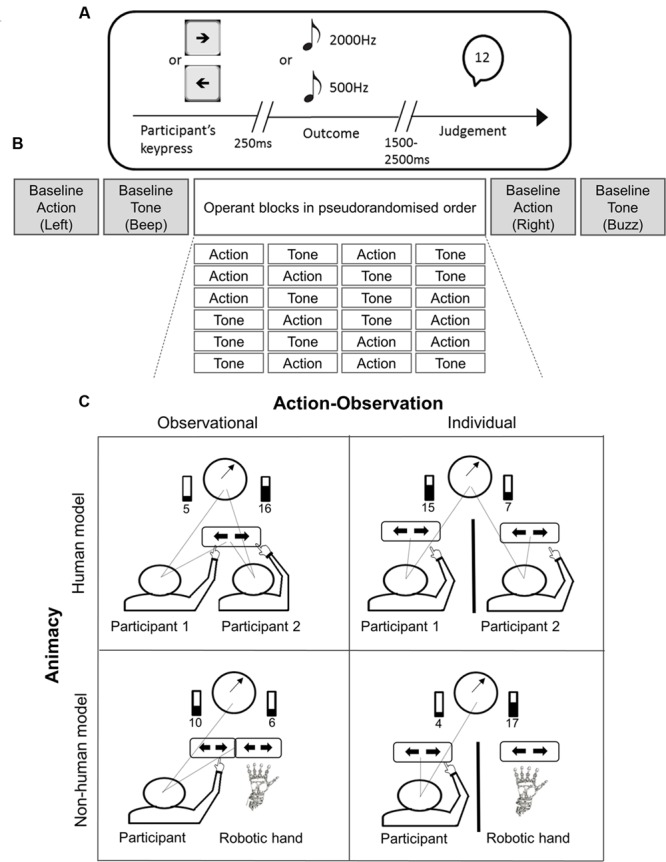
**(A)** Timeline of an experimental trial. Participants were instructed to press the left or right arrow keys at a time of their own free choice. In the operant conditions, each keypress was followed by a beep (2000 Hz) (rewarded) or a buzz (500 Hz) (non-rewarded) after 250 ms with different probabilities. At the end of the trial, participants reported the perceived time of the action or of the tone. See the text for full explanation. **(B)** The experimental session started with one Baseline Action and one Baseline Tone block. Four main experimental blocks, 2 Operant-Action and 2 Operant-Tone then followed in pseudorandomised order. All possible block orders are shown. Each pair performed one of the six possible orders. The order of the operant blocks was counterbalanced across participants. The session ended with the execution of the complementary blocks of Baseline Action and Baseline Tone. **(C)** 2 × 2 factorial design with the factors of animacy (human/robot model) and action-observation (observational/individual). In the human-model groups two participants were seated in front of the screen and alternated in performing trials. One of the participants was replaced with a robotic-hand in the non-human groups. An opaque barrier (thick black line) further divided each group to ‘observational’ and ‘individual’ groups. See the text for full explanation. Progress bars displayed the cumulative total of rewarded trials. Dotted lines represent gaze direction.

The beep was always defined as the “correct” tone, and was associated with a monetary reward (£0.10 per trial, paid at the end of the experiment). Participants were not rewarded for key presses that produced buzzes. After the clock hand stopped, participants judged the clock hand position at the moment of pressing the key. Finally, the *operant tone* condition was similar to the operant action condition, with the difference that participants had to judge the clock hand position at the time of the tone onset (beep or buzz) (**Figure 1A**).

The order of blocks was as follows. Baseline action and baseline tone conditions were tested in separate blocks of 40 trials at the beginning and end of the experiment, sandwiching the operant conditions. The order of baseline blocks was randomized. Each operant condition was tested in two blocks of 40 trials. Therefore, each experimental session consisted of four operant blocks in a random order, bracketed by four baseline blocks (**Figure 1B**).

### Experimental Design

**FIGURE 2:**
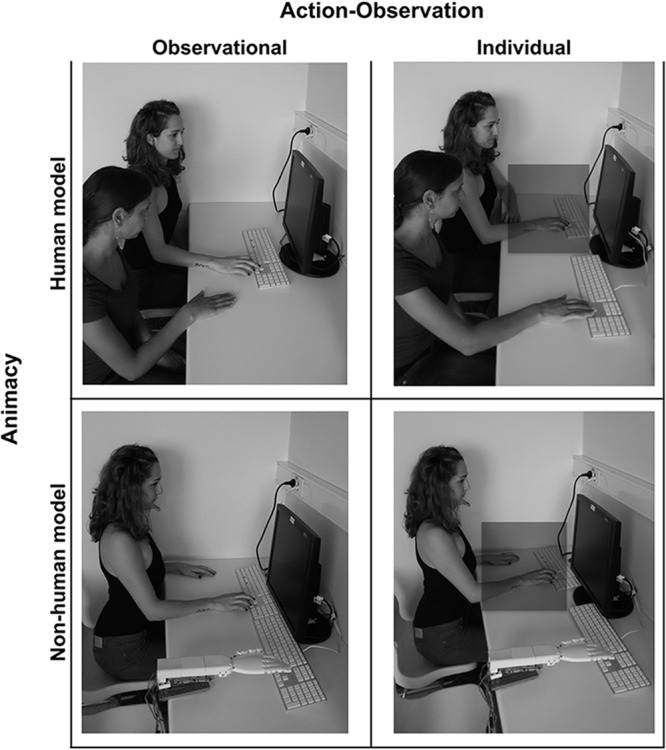
**2 × 2 factorial design with the factors of animacy (human/robot model) and action-observation (observational/individual).** See **Figure 1C** for details.

The study used a 2 × 2 between-subject design. The first factor, *animacy*, was related to the type of model available for action observation. Participants were randomly assigned to either a *human model* group, or a *non-human* (robotic hand) *model* group. The second factor, *action-observation*, related to the availability of information from the model. Participants were randomly assigned to either *observational*, or *individual* groups (see later) (**Figures 1C** and **2**).

In the *human model* groups, participants were invited to the experiment in gender-matched pairs. Each pair was seated in two chairs positioned next to each other in front of the screen. In three cases where one invited participant did not attend, he/she was replaced by a gender-matched assistant who was blind to purpose of the study. Data from the assistant was not included in the analysis. At the beginning of each block, the participant sitting on the left side started the task. The two participants then alternated in performing the rest of the trials. Alternating in performing the trials aimed to keep participants engaged with the task and to encourage them to constantly follow each other’s action. Throughout the task, participants had to judge their *own* actions and outcomes, only. In the *individual* group, a barrier was placed between the participants, so that they could not see each other’s actions but they could see and hear each other’s outcomes. Thus, the information from the other’s actions was irrelevant for selecting the appropriate action, because action-outcome mappings for others’ actions were not clear. This barrier was removed for the *observational* group, so each participant could see and hear each other’s actions and outcomes. The behavioral task and the order of the blocks were exactly the same for both groups. To be certain that participants paid attention to each other’s actions in the observational group, participants were occasionally asked to report the action (which key was pressed) and the outcome (beep or buzz) of their partner in the immediately preceding trial. They lost a point (£0.10) for giving incorrect answers. The cumulative total of rewarded trials (i.e., actions producing beep, rather than buzz) was displayed on the screen after each trial, and participants were paid a bonus at the end of the experiment based on the total number of rewarded trials.

In the *non-human model* groups, the co-participant was replaced with a robotic hand. Thus, participants participated singly, rather than in pairs. They were instructed that the robotic hand was performing the same task, and robotic trials would alternate with their own. Two keyboards were placed both in front of the participant and in front of the robotic hand. The index and ring fingers of the robotic hand were aligned with the same keys as for the participant’s hand. The robot hand was anthropomorphic and was based on rapid servo-actuated tendons rotating each finger joint. In fact, the robot hand movement was triggered by an invisible experimenter, wearing a dataglove to which the robot was slaved, and located behind a partition. The ‘judgments’ of the robot hand, were in fact entered by the experimenter. The experimenter participated in the task on the same basis as a participant in the experiment, but her data was not analyzed. Importantly, in a previous experiment, sense of agency was not influenced by the irrelevant movement of the robotic hand. No difference in time judgment was observed in a condition where the robotic hand made intermediate, but irrelevant movements between the participant’s action and the tone, and a condition without the robotic hand (Caspar et al., 2015). This suggests that noise generated by the robotic hand is unlikely to represent a confounding influence on time judgement. In the *observational* group, the participant thus saw the robot performing human-like actions. As in the *human model* group, participants were occasionally asked to report the action and the outcome of the robot in the immediately preceding trial. In the *individual* group, the participant was only able to see the judgments corresponding to the robot hand movement, displayed on the monitor, but the robot hand itself was hidden from view by a barrier, as in the *human model* group. All participants saw how the robotic hand moved before starting the experiment, and could hear the motor noise associated with each movement of the robot during the whole experiment. To make the robot’s performance seem identical to a human agent, participants were told that the robotic hand is performing the same task as the participant and will make its own decisions whether to press the left or right arrow key. However, it does not make perfect decisions and may make mistakes from time to time. The behavioral task and the order of the blocks were exactly the same for both groups.

### Data Analysis

For each trial, *judgment error* was defined as the difference between the judged clock hand position and the actual onset of the corresponding event. A positive judgment error indicated a perceptual delay; a negative judgment error an anticipation. The mean and standard deviation of the judgment errors across trials were then measured for each block type (see **Figure 1B**). Action binding was defined as the shift of reported time of action toward its outcome, and was calculated by subtracting each participant’s judgment mean error in the *baseline action* conditions from that in the *operant action* condition. Likewise, tone binding was defined as a shift in the perceived time of a tone toward the action that triggered it. Tone binding was calculated by subtracting each participant’s judgment error in *baseline tone* conditions from *operant tone* condition. Thus, perceptual association of an action with a subsequent tone produced a positive value for action binding, and a negative value for tone binding. To investigate continuous acquisition of sense of agency, we considered how the action and tone binding effects changed over successive trials. The relation between binding effect and trial number was calculated within each group using a linear regression approach. As choice of bin size would be rather arbitrary, the regression slope was computed against single trials rather than trial bins. Importantly, because timing estimates using the rotating clock hand were noisy, we used bisquare robust regression analysis to avoid overall estimates of changes in binding being excessively driven by a small number of trials with large judgment errors toward the end or beginning of the experimental session (Huber, 2004).

Our experimental design was based on the 2 × 2 factorial combination of groups (**Figures 1C** and **2**). We had clear directional hypotheses about the differences between groups, based on previous action observation literature. Specifically, we predicted that participants would show greater context-related changes in intentional binding, in observational rather than individual groups, and for a human model, rather than a non-human model. Because we did not have prior hypotheses regarding which combination of our two dependent variables (action binding and tone binding) might be influenced by social context, we applied 2 × 2 multivariate analysis of variance (MANOVA) applied to both action and tone binding simultaneously. Inspection of canonical variates for significant MANOVA effects was used to investigate the extent to which our experimental design factors influenced each dependent variable, and these influences were confirmed with univariate *t*-tests.

## Results

Seventy two participants were tested in total. Eight were excluded because of technical errors (two participants) or inability to complete the task (six participants). Data from 64 participants were retained for analysis (human model observational group, 17 participants; human model individual group, 16 participants; non-human model observational group, 16 participants; non-human model individual group, 15 participants).

We confirmed an overall effect of action binding and tone binding, relative to the respective baseline conditions (**Table 1**). The perceived time of action execution was shifted toward the tone (outcome) by an average 66 ms [*SE* = 9 ms, *t*(63) = 7.50, *p* < 0.01 one-tailed comparison against zero]. The perceived time of tone was shifted toward the action by an average -105 ms [*SE* = 13 ms, *t*(63) = -8.00, *p* < 0.01 one-tailed comparison against zero]. These results replicated previous reports on intentional binding (Haggard et al., 2002a,b).

**Table 1:** The mean (and standard error across participants) for binding, and robust regression slope showing the relation between binding and trial number.

Groups	Mean binding (ms)		Regression slope (ms/trial)	
	Action binding	Tone binding	Action binding	Tone binding
Human observational	64 (17)^∗^	-133 (25)^∗^	1.9 (0.5)^∗^	2.1 (0.9)
Human individual	71 (17)^∗^	-100 (27)^∗^	-0.9 (0.8)	1 (0.9)
Robot observational	63 (18)^∗^	-85 (27)^∗^	1 (0.4)	-0.8 (0.8)
Robot individual	67 (20)^∗^	-98 (24)^∗^	0.2 (0.4)	1 (0.7)

*Separate one-sample, one-tailed *t*-tests were used to investigate each dependent variable. ^∗^Significant values after Bonferroni correction for four conditions.*

We next investigated possible differences in binding between the 4 groups of the 2 × 2 design. There was no significant main effect of *animacy* [*F*(2,59) = 1.00, *p* = 0.37, η^2^ = 0.03] or *action-observation* [*F*(2,59) = 0.07, *p* = 0.93, η^2^ < 0.01] and no interaction [*F*(2,59) = 0.63, *p* = 0.54, η^2^ = 0.02] on the combination of both mean action and tone binding. Therefore, no follow-up analyses were performed on mean binding data.

**FIGURE 3:**
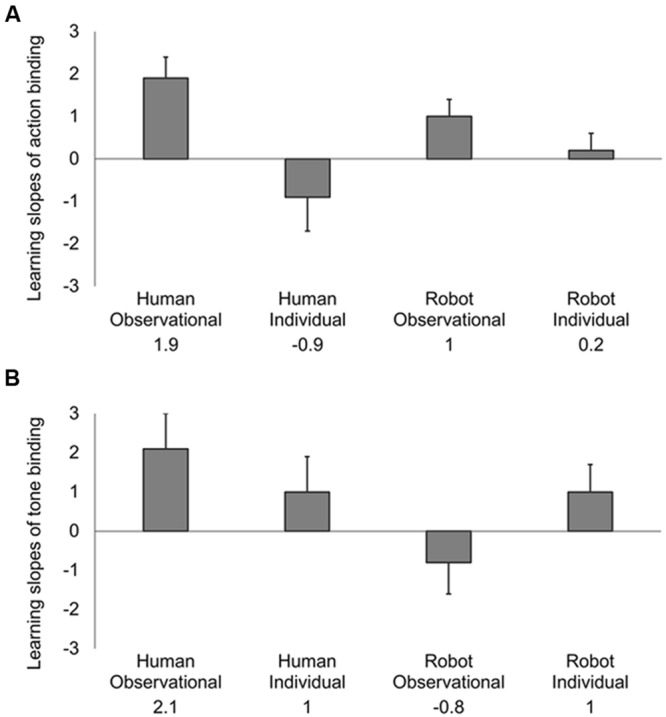
**Changes of intentional binding over trials, plotted separately for **(A)** action binding and **(B)** tone binding.** The values on vertical axes are regression slopes (changes of binding in ms per trial). A positive slope for this regression would correspond to an increase in action binding with progressive exposure to the other’s action, but a decrease in tone binding, because of the negative sign of tone binding. Error bars show standard errors.

To investigate how these contextual factors might progressively transform intentional binding, separate robust regressions of action and tone shift against trial number were fitted for each participant. A positive slope for this regression would correspond to an increase in action binding with progressive exposure to the other’s action. Conversely an increase in tone binding with exposure to the other’s action would produce a negative slope, because of the negative sign of tone binding (see Supplementary Figure S1). We calculated the slope for each participant (i.e., random effects). Next, the robust regression slopes for action and tone binding were used as dependent variables in MANOVA. The model revealed a significant intercept [*F*(2,59) = 3.70, *p* = 0.03, η^2^ = 0.11], implying a progressive linear change in combined action and tone binding during each block (**Table 1**). Having established evidence for progressive changes in intentional binding over the whole dataset, we next investigated whether these effects varied across the different experimental groups. MANOVA showed a significant main effect of *action-observation* on combined action and tone binding slopes [*F*(2,59) = 4.73, *p* = 0.01, η^2^ = 0.14]. Separate univariate tests revealed a significant main effect of *action-observation* on action binding slope [*t*(62) = 3.13, *p* < 0.01, *d* = 0.80] but not on tone binding slope [*t*(62) = -0.31, *p* = 0.76, *d* = 0.08]. This implied that the major social effect of observing others’ actions was a change in action binding (**Figure 3**). These impressions were confirmed by the standardized canonical coefficients from the MANOVA of robust regression slopes. These suggested that observation primarily influences action binding (standardized coefficient 1.07) rather than tone binding (standardized coefficient -0.07, see Supplementary Table S1 for coefficients for the full MANOVA design). We found no significant MANOVA main effect of the *animacy* factor [*F*(2,59) = 1.46, *p* = 0.24, η^2^ = 0.05], suggesting that animacy had no prominent effect on changes of intentional binding. The interaction between these two factors approached but did not reach the level of significance [*F*(2,59) = 2.72, *p* = 0.07, η^2^ = 0.09]. This suggests that contrary to our primary prediction, the effect of action-observation on acquisition of sense of agency is not specific to observing human agents.

We also tested the intercepts of the robust regression, to assess whether there were differences in the initial level of intentional binding in each condition, in addition to the changes in binding captured by our test of regression slopes. We found no main effect of action-observation [*F*(2,59) = 1.96, *p* = 0.15, η^2^ = 0.06], or animacy [*F*(2,59) = 2.27, *p* = 0.12, η^2^ = 0.07] factors, and no significant interaction between them [*F*(2,59) = 1.14, *p* = 0.33, η^2^ = 0.04]. This suggests that the observed effect is driven by a process that cumulates gradually during the experiment, as a function of exposure to others’ actions, and does not merely reflect a difference in starting point.

To check whether the observed increase in perceptual delay over operant trials is actually driven by the social context, and not by some general feature of the task, we checked the slopes in the baseline condition. Separate one-sample, one-tailed *t*-tests did not show any relation between binding and trial number in the baseline condition (Supplementary Table S2). This suggests that non-specific changes in perceptual delay, related to factors other than social-observational context, cannot account for the progressive changes in intentional binding seen in our experimental conditions.

**FIGURE 4:**
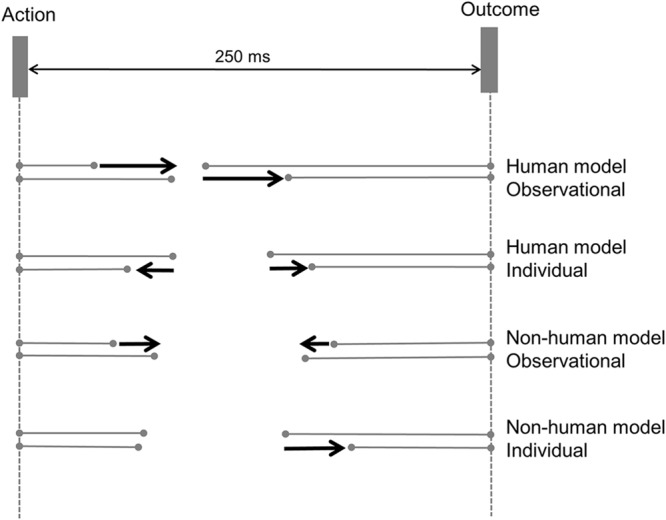
**The evolution of action and tone binding, plotted separately for each experimental group.** In each group, average binding in the first and last 10 trials is shown in the upper and lower lines, respectively (lines with endpoints). Thick black arrows illustrate the change in action and tone binding over successive trials. A rightward arrow corresponds to an increase in action binding, but a decrease in tone binding. A leftward arrow corresponds to a decrease in action binding, but an increase in tone binding. Binding effects are drawn to scale.

To summarize, we observed a pattern of progressive change in implicit measures of sense of agency based on time perception. MANOVA testing confirmed the simultaneous presence of two progressive changes over the course of each block. First, outcome perception became increasingly accurate, leading to a reduction in tone binding. Second, the perceived time of action was progressively captured by the outcome. This latter effect was most marked for observing others’ actions rather than individual performance, and was detected when observing another person, as well as observing a human-like robot (**Figure 4**).

## Discussion

We used a simple experimental design to investigate the influence of observing others’ actions on an implicit measure of sense of agency. We also investigated whether a human model was necessary for these effects to emerge. We found that the perceived time of action and tone both moved toward the actual time of outcome in the observational group. This produced a progressive increase in action binding, and a progressive decrease in tone binding, with cumulating experience. Interestingly, this shift in the perceived time of action and tone was detected in participants who observed a human model as well as in participants who observed a robot hand, although it was numerically stronger in the former group. Thus, our results strongly suggest that the sense of agency for instrumental action, at least as measured with intentional binding, is not stationary, but gradually changes based on evidence both internal and external to the agent. This development of sense of agency is not restricted to observing a human agent. MANOVA analysis of robust regression slopes clarified the direction of this influence of social context: action binding increased across successive trials, while tone binding decreased. That is, with repetition, the perception of actions became increasingly biased toward the outcomes of those actions, while the perception of the outcomes themselves became progressively less biased.

These changes suggest an ‘ideomotor shift’ in the perception of instrumental actions, with exposure leading to an increased importance of the outcome, relative to the action that produces it. Initially, the action itself has a relatively strong weighting in computing the sense of agency. However, repeated observation of others executing actions, and producing the corresponding outcomes, induced a gradual shift in the weighting of experience away from the action, and toward the outcome. These progressive changes in binding were stronger when participants could observe the actions of the other agent than when they could not. Further, the effects of observing a human confederate were not statistically different from observing a robot hand. These results are also compatible with a recent framework derived from theory of event coding. Based on this framework, in a social context where two participants perform in a joint response task, actions and representations of each participant can reshape the representations of the other’s actions (Prinz, 2015). We similarly showed that observing the actions of another agent reshapes the salience of one’s own action outcomes. Finally, these effects of observation were found primarily in action binding. Tone binding was essentially similar between the different observation groups, and showed only a general trend in all conditions toward less biased tone perception with repeated exposure. In contrast, observing the instrumental actions of another agent led to the perception of one’s own actions being more strongly captured by the outcome.

This pattern of results is consistent with the concept that observing the instrumental actions of another agent may enhance the salience and importance of action outcomes in producing sense of agency, as measured by temporal event perception. We could not measure saliency of events directly in our design, but salient events are known to capture temporal event perception (Wolpe et al., 2013). Importantly, the intentional binding measure that we used as a proxy for sense of agency was entirely independent of the action selection task, and we gave no feedback regarding timing judgments. Thus, the changes in the experience of instrumental action were incidental to the participant’s primary task of identifying which key to press on each trial. The observed actions of the other agent were, moreover, irrelevant to the participant’s secondary task of timing judgment, because participants only judged the time of their own actions or outcomes. For these reasons, the social influence on sense of agency appears to be an incidental and automatic effect of social context. That is, simply having access to information about the relation between others’ actions and outcomes produced a stronger representation of the outcomes of one’s own instrumental agency. This result suggests a social facilitation of sense of agency.

In this study, participants alternated between performing the task and observing their partner, meaning that they were immersed in a mixture of observational and performance environments. Therefore, we cannot distinguish the pure effects of observation in the absence of any action at all. Rather, our findings suggest that observation can enhance one’s own sense of agency during performance. In that sense, our experimental design involved a kind of intermittently active observation, rather than purely passive observation. Merely watching, without ever doing, might not have any consequences for subsequent agency. For example, in a more classical observational learning paradigm, participants learned the association between an action and its effect by observing a model. In that study, learning a novel action-effect association occurred strictly through observation of another agent (Paulus et al., 2011), and without any action on the agent’s part. However, that study did not address the sense of agency directly.

Most previous studies on sense of agency have not addressed the process of acquisition of agency. This may be because human instrumental learning is often very rapid. Moore and Haggard (2008) showed that reinforcement of action-outcome association on the immediately preceding trial can increase action binding compared to an experience in the more distant past. Another study showed that intentional binding depends on predictions of when the outcome will occur, based on previous experience (Walsh and Haggard, 2013). Interestingly, these temporal predictions were updated very rapidly, over a few trials. Our results point to the acquisition of agency over a slower timescale of several trials. This difference in timescales could reflect either a different mechanism of acquisition of agency from one’s own vs. others’ actions, or a difference in acquisition rate.

Contrary to our primary prediction we did not find any significant difference between observing a human and a non-human agent. At least one previous study has explored awareness of actions in human and non-human agents. Participants reported the perceptual time of action that was executed by themselves, by another participant, or by a machine. No difference was observed between the judgements for self- and other-generated actions, but a significant difference was detected for machine actions. Participants attributed their intentions to others, but not to the machine (Wohlschläger et al., 2003). That experiment, however, differed from ours in that participants had to make agency judgments for their own and other-generated actions while in our study they had to make judgments only for their own actions. The joint action literature suggests that similarity between agent and co-agent can influence action co-representation as measured by joint Simon effect (JSE) (Dolk et al., 2014). Interestingly, when participants shared a task with a humanoid robot, their belief about the humanness of the robot influenced JSE. Human agents co-represented the action of the robot when sharing the task with a human-like but not a machine-like robot (Stenzel et al., 2012). In another experiment, JSE was observed with both biological and non-biological co-actors. The authors suggested that JSE can occur as long as the participant believes that they are interacting with another agent, regardless of the agent’s identity (Wen and Hsieh, 2015). In our experiment, the robot hand was anthropomorphic. Participants were told that the robotic hand makes its own decisions but can also make mistakes in a very human-like manner. We believe that the comparable effects for human and robotic hands may be due to anthropomorphising the robotic hand: participants were told that they are interacting with an ‘intentional,’ ‘human-like’ agent (Epley et al., 2007). Interestingly, even though the robotic hand was life-like, it was obviously not connected to a body. This suggests that the influence of physical appearance of the robot on action representation was overwritten by top–down belief processes (Stenzel et al., 2012). Therefore, observing robots controlling external events may similarly play an important role in mentally representing the impact of our own actions, when one *believes* that the robot functions in a human-like manner. Future research might systematically manipulate the factors that make an artificial agent sufficiently human-like.

Previous studies using intentional binding effects have used a range of dependent variables. One approach is to simply sum the action binding and tone binding to create an overall binding score. We have not used that measure here. Dissociations between action binding and tone binding have been reported previously (Wolpe et al., 2013). Instead, our main approach involved analyzing action binding and tone binding in parallel, using a multivariate approach. In addition, our analysis of the effects of observing other agents focussed on progressive changes in temporal judgment over repeated trials, while almost all previous studies analyzed means. Because action binding and tone binding are measured in separate blocks, it is not possible to calculate a composite binding measure for individual trials. We therefore preferred to quantify changes in action and tone binding separately and then apply MANOVA to both measures simultaneously.

We recognize a number of limitations in our study. First, although the number of participants is high, the design may seem underpowered for between-subject comparison, since there are just 16 people per group. However, other studies on joint-action, which is the most relevant comparable field, have used similar group sizes (Obhi and Hall, 2011a; van der Wel et al., 2012; Stenzel et al., 2014; Eskenazi et al., 2015). Another limitation is the position of response pads. In the robot observational condition, the robot and the human participants used separate response pads. This was a technical limitation: the robot hand did not have sufficient range of movement for us to move it away from the response key while the participant made their action. In contrast, for the human observation condition, the model and the participant took turns to use the same response pad. We acknowledge this as a potential confound. Nevertheless, the relevant detail for the task was that both human and robot could produce outcomes by pressing one of the two keys.

Overall, we suggest that presence of others increases the role of external outcomes in action awareness. As social and communicative animals, a dominant source of our information about the external world is not our own direct experience, but knowledge acquired from others. That is, social communicative situations provide an opportunity to acquire beliefs about the world (Grice and Strawson, 1956). We believe that our findings highlight the crucial role of directing attention toward the actions and outcomes of others in sociocultural transmission of skills in humans. According to one view, social responsibility for action involves a self-to-other direction of cognitive generalization. That is, one first represents the consequences of one’s own instrumental actions on the immediate physical environment, and, in a second stage, represents that one’s actions also have consequences on other people. An alternative view suggests an other-to-self direction: representing the actions of others may facilitate representation of the impact of what one does oneself (Hauf and Prinz, 2005). Our results suggest an important role of other-to-self, or me-like-you processes in human sense of agency. When other agents are present in a social context, actions become progressively represented in terms of their external effects, rather than the intentional processes associated with execution. Importantly, this change is not human-specific and also occurs in the presence of human-like non-biological objects. This is particularly important given the ever increasing interaction between humans and technology (for a review see Limerick et al., 2014). We speculate that contexts in which other agents are present lead to an emphasis on the external, shared, consequences of action, rather than individual intentionality.

## Author Contributions

NK developed the study concept. NK, BB, and PH designed the study. NK and EC ran the experiment. NK and PH analyzed the data. NK, BB, EC, and PH helped with interpretation of the data. NK and PH drafted the manuscript. BB and EC revisited the manuscript for intellectual content. NK, BB, EC, and PH approved the version of the manuscript to be published.

## Conflict of Interest Statement

The authors declare that the research was conducted in the absence of any commercial or financial relationships that could be construed as a potential conflict of interest.
