# Supplementary material for: Social Transmission of Experience of Agency: An Experimental Study
Source: Front Psychol. 2016 Aug 30;7:1315. doi: 10.3389/fpsyg.2016.01315 (PMC5003881; doi:10.3389/fpsyg.2016.01315)
Supplement: Supplementary file 1 [file Table_1.DOCX]

Supplementary Table 1. Standardized discriminant coefficients from MANOVA of robust regression slopes. Bold lettering indicates significant MANOVA effects. Intercept: action binding and tone binding show comparable change across trials. Observation primarily influences action binding.

|  | Action binding | Tone binding |
| --- | --- | --- |
| Intercept | **0.761** | **0.757** |
| Main effects  observational vs. individual | **1.066** | **-0.072** |
| Main effects  Human model vs. non-human model | -0.105 | 1.013 |
| Interaction | 0.764 | 0.754 |
