# Supplementary material for: Social Transmission of Experience of Agency: An Experimental Study
Source: Front Psychol. 2016 Aug 30;7:1315. doi: 10.3389/fpsyg.2016.01315 (PMC5003881; doi:10.3389/fpsyg.2016.01315)
Supplement: Supplementary file 2 [file Table_2.DOCX]

Supplementary Table 2. The mean (and standard error across participants) for robust regression slope showing the relation between binding and trial number in baseline trials. Separate one-sample, one-tailed t-tests were used to investigate each dependent variable.

| Groups | Regression slope (ms/trial) | |
| --- | --- | --- |
|  | Baseline Action | Baseline Tone |
| Human observational | 0.0 (0.6) | 0.5 (0.5) |
| Human individual | -0.4 (0.6) | -0.3 (0.4) |
| Robot observational | -0.4 (0.4) | 0.1 (0.3) |
| Robot individual | -0.1 (0.3) | 0.3 (0.4) |
