# Supplementary figures and images for: Social Transmission of Experience of Agency: An Experimental Study
Source: Front Psychol. 2016 Aug 30;7:1315. doi: 10.3389/fpsyg.2016.01315 (PMC5003881; doi:10.3389/fpsyg.2016.01315)

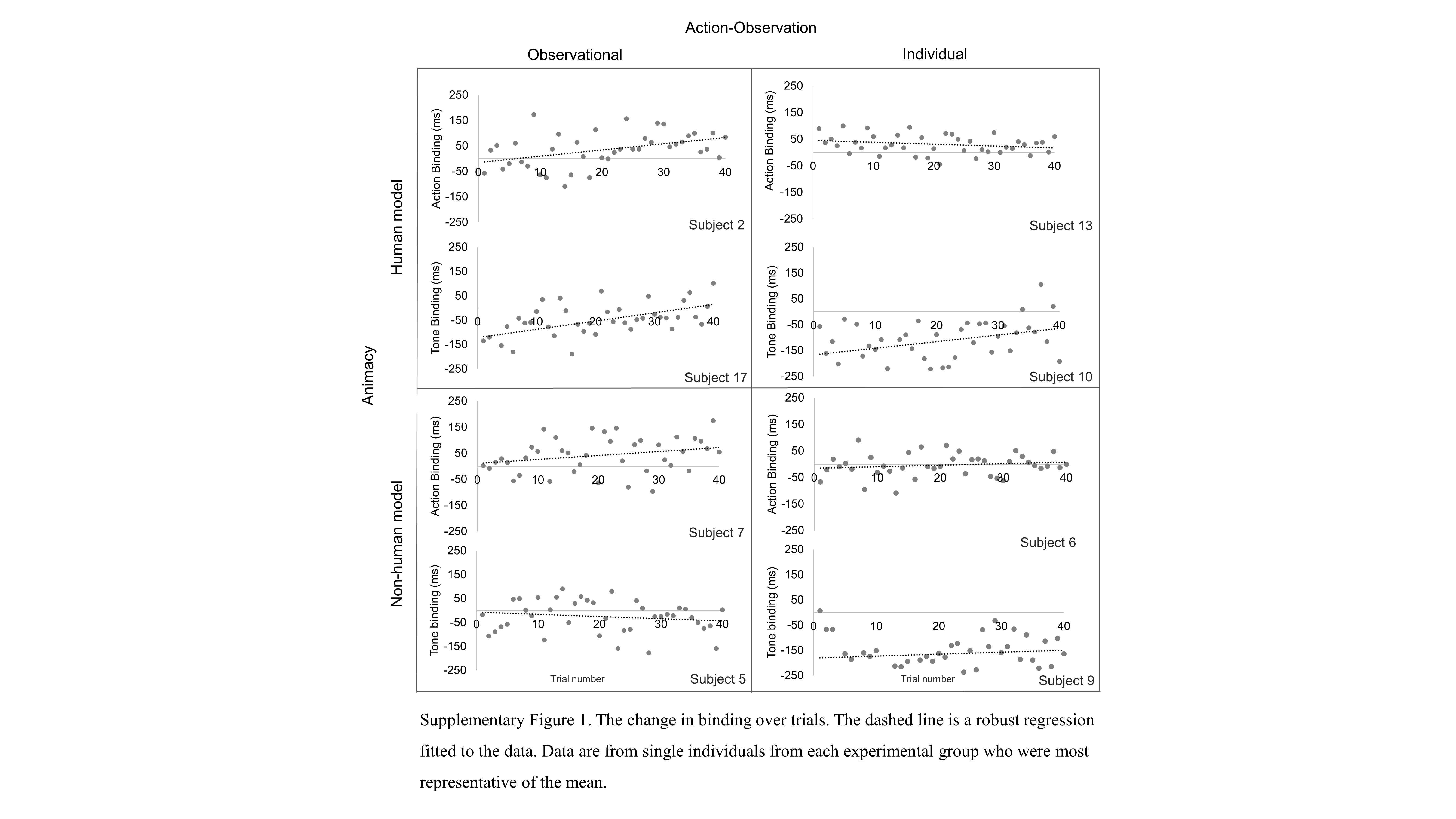

Supplement: Supplementary file 3 [file Image_1.TIF]
